# Ambient Fine Particulate Matter Exposure and Risk of Cardiovascular Mortality: Adjustment of the Meteorological Factors

**DOI:** 10.3390/ijerph13111082

**Published:** 2016-11-04

**Authors:** Kai Luo, Wenjing Li, Ruiming Zhang, Runkui Li, Qun Xu, Yang Cao

**Affiliations:** 1 Department of Epidemiology and Biostatistics, Institute of Basic Medical Sciences Chinese Academy of Medical Sciences, School of Basic Medicine Peking Union Medical College, Beijing 100005, China; kevinluopumc@hotmail.com (K.L.); liwenjing197@163.com (W.L.); zrm2018@126.com (R.Z.); 2 Center of Environmental and Health Sciences, Chinese Academy of Medical Sciences, Peking Union Medical College, Beijing 100005, China; 3 College of Resources and Environment, University of Chinese Academy of Sciences, Beijing 100049, China; 4 State Key Laboratory of Resources and Environmental Information System, Institute of Geographic Sciences and Natural Resources Research, Chinese Academy of Science, Beijing 100101, China; 5 Clinical Epidemiology and Biostatistics, School of Medical Sciences, Örebro University, Örebro 70185, Sweden; yang.cao@ki.se; 6 Unit of Biostatistics, Institute of Environmental Medicine, KarolinskaInstitutet, Stockholm 17177, Sweden

**Keywords:** fine particulate matter, PM_2.5_, mortality, time stratified case-crossover study, distributed lag nonlinear model

## Abstract

Few studies have explicitly explored the impacts of the extensive adjustment (with a lag period of more than one week) of temperature and humidity on the association between ambient fine particulate matter (PM_2.5_) and cardiovascular mortality. In a time stratified case-crossover study, we used a distributed lag nonlinear model to assess the impacts of extensive adjustments of temperature and humidity for longer lag periods (for 7, 14, 21, 28 and 40 days) on effects of PM_2.5_ on total cardiovascular mortality and mortality of cerebrovascular and ischemic heart disease and corresponding exposure-response relationships in Beijing, China, between 2008 and 2011. Compared with results only controlled for temperature and humidity for 2 days, the estimated effects of PM_2.5_ were smaller and magnitudes of exposure-response curves were decreased when longer lag periods of temperature and relative humidity were included for adjustments, but these changes varied across subpopulation, with marked decreases occurring in males and the elderly who are more susceptible to PM_2.5_-related mortalities. Our findings suggest that the adjustment of meteorological factors using lag periods shorter than one week may lead to overestimated effects of PM_2.5_. The associations of PM_2.5_ with cardiovascular mortality in susceptible populations were more sensitive to further adjustments for temperature and relative humidity.

## 1. Introduction

Interest in the adverse effects of particulate matter with aerodynamic diameter ≤2.5 μm (PM_2.5_) has greatly increased because of its multiple sources and the varied toxicity of the components, as well as the small size of PM_2.5_ that can facilitate it being inhaled into the deep lung and then triggering adverse direct and indirect effects on the cardiopulmonary system [1,2]. Mounting evidence has proved that both short- and long-term exposure to PM_2.5_ can contribute to elevated risk of overall cardiopulmonary morbidity and mortality [3,4,5,6,7].

Despite this evidence, times series studies on the acute effects of PM_2.5_ and other pollutants still face the challenge to adequately controlling the potential confounding that originates from unmeasured and measured factors. However, the confounding control when assessing the acute mortality effects of PM_2.5_ mainly focus on the adjustment for the unmeasured confounding factors, such as seasonal or long-term time trends [5,8,9,10]. Indeed, meteorological factors, especially temperature and relative humidity, are typically considered to be potential confounders of the acute effects of PM_2.5_ and other pollutants [11]. Furthermore, evidence has shown that temperature effects on mortality were more prolonged and stronger than those of air pollutants [12,13]. Accordingly, if the effect of temperature was only controlled for a short lag period (within one week), this might raise concerns about whether the previously reported acute mortality effects of PM_2.5_ were the result of residual confounding by inadequately controlled meteorological factors [14], especially for the previous Chinese studies where the effect of temperature was adjusted for a few days, or even only for the current day [8,10,15]. Thus, further evidence is need to address this concern and to demonstrate the explicit impact of adjusting for temperature and other meteorological factors for longer lag period on PM_2.5_ effects.

In addition, apart from the accumulation of evidence of the effects of air pollutants on non-accidental mortalities, exploring exposure-response relationships plays a critical role in the quantitative assessment of air pollutants’ hazards and the estimation of attributable death [16]. Extending the exploration of impacts of adjustment of temperature and other meteorological factors on effect estimations of PM_2.5_ to relevant exposure-response relationships can provide a full picture of the impacts of longer lag period adjustments of those weather factors on the association between PM_2.5_ and mortality. Furthermore, the analysis in varied susceptible populations can also enhance the understanding of the exposure-response relationship for mortality associated with PM_2.5_ for different subpopulations (e.g., the elderly population).

With the above considerations in mind, we conducted the present study to investigate the potential impacts of extensive adjustments of temperature and relative humidity with accounted lag periods longer than one week on the associations and corresponded exposure-response relationships of cardiovascular mortalities, including total cardiovascular disease (CVD), cerebrovascular disease (CBD) and ischemic heart disease (IHD) mortality, for PM_2.5_ in both the whole population and relevant subgroups.

## 2. Material and Methods

### 2.1. Study Setting

Beijing, the capital of China, is located in the northwest of Beijing-Tianjin-Hebei delta, which is surrounded by the Yan Mountains in the west, north and northeast direction, and is the nation’s political, cultural and educational center. There are 16 administrative districts in Beijing and the average population for the study period from 2008 to 2011 was 19.02 million [17].

### 2.2. Data Collection

For the present 2008–2011 study period, we collected population-based daily mortality data for all the 16 urban and suburban administrative districts in Beijing from the Causes of Death Registry of Chinese Center for Disease Control and Prevention (China CDC). The causes of death were coded according to the International Disease Classification Codes, version 10 (ICD-10). Deaths for CVD (I00–I99), CBD (I60–I69), and IHD (I20–I25) were examined.

Because PM_2.5_ was not yet routinely monitored in China until late December, 2012, we obtained hourly concentrations of PM_2.5_ published by the air quality monitoring station of the U.S. embassy in China, which is located in the Chaoyang district of Beijing (see Figure S1 in Supplementary Materials). Regular monitoring at the U.S. embassy began in April, 2008 and the monitoring radius of the station is approximately 40 km, covering nearly 79.2% of Beijing’s total population and all areas with dense (>5000 people/km^2^) population [18]. Recent studies have made a comprehensive comparison of the PM_2.5_ data quality of both from the U.S. embassy station and Chinese national monitoring stations in Beijing and concluded that PM_2.5_ data from the two sources were highly consistent in both yearly and seasonal trends, especially in the urban districts around the U.S. embassy [19], which proved the reliability of the PM_2.5_ data from the U.S. embassy. The daily average concentrations were calculated when the cumulative time of hourly PM_2.5_ concentration was more than 12 h per day, otherwise, the daily concentration was regarded as missing.

Data of daily mean temperature and relative humidity was retrieved from the Beijing Observatory (Station No.: 54511) of the China Meteorological Administration, which is located in the Daxing district in Beijing. Other daily data of meteorological factors, including barometric pressure and wind, were also collected from the same station.

Because there were some missing values in several days for our obtained PM_2.5_ data, listed in Supplementary Materials Table S1, we developed a series generalized additive models based on the relationships between daily PM_2.5_ concentration and meteorological variables to determine those missing values rather than simply excluded them, by which the impacts of those missing values on mortality effects of PM_2.5_ could be reduced [20]. A detailed description of the statistical methodology for the imputation models is also provided in Supplementary Materials, in which Figure S2 displays the performance of imputation model.

### 2.3. Statistical Analysis

#### 2.3.1. Analytic Plan

We first estimated the associations between PM_2.5_ and cardiovascular mortality. The corresponding exposure-response relationships with the effect of temperature and humidity were controlled for current and previous day. This modeling strategy was commonly adopted in previous studies (see Supplementary Materials Table S2) and the results were used as the basis for the subsequent comparison. We then performed the analysis using a series of extensive adjustments of temperature and humidity, and the results from this stage were compared with the base results. In particularly, longer lag periods of effect of temperature/humidity including 7, 14, 21, 28 and 40 days were used for the extensive adjustments.

#### 2.3.2. Associations and Exposure-Response Relationships between PM_2.5_ and Mortality

The associations between PM_2.5_ and CVD,CBD and IHD mortality were investigated using a time stratified case-crossover (TCCO) design [21], and the conditional logistic regression used in this study design can be regard as a special case of time series Poisson regression with indicator of “strata” [22]. This equivalence allows more flexible analysis methods to be applied to the TCCO design [23]. The over-dispersion in the original daily counts can be accounted for by assuming the daily counts follow a quasi-Poisson distribution [24]. Thus, we adopted the Poisson regression model that allowed for over-dispersion based on time series data combined with a distributed lag-nonlinear model (DLNM) [25], which has been elaborated elsewhere [26]. This approach can facilitate the extensive adjustment of the long-term cumulative nonlinear effects of temperature and relative humidity on mortality [12], consequently, reducing the potential residual confounding of weather [14] and the effects of PM_2.5_ will be more easily examined with this approach than conventional TCCO analysis [25]. In the primary analysis, we adopted a fixed and disjointed time window, i.e., calendar month, to control for long term and seasonal time trends. The confounding effects of the day of week were controlled as a categorical term of “Dow”. For comparison, the effect of weather factors was controlled using the common strategy in large multicity studies [5,27]. Specially, temperature, relative humidity and barometric pressure were controlled using natural cubic splines with a degree of freedom (DF) of three for two days (current day and previous day). We firstly built a “primary adjusted model” that allows for potential confounders when estimating PM_2.5_ effects. The model is expressed as:

(1) $$\log\left(y_{t}\right)=\alpha +{\eta \mathrm{Strata}}_{t}+{\gamma \mathrm{Dow}}_{t}+\mathrm{ns}\left({\mathrm{mhumd}}_{01},3\right)+\mathrm{ns}\left({\mathrm{mpressure}}_{01},3\right)+\mathrm{ns}\left({\mathrm{mtemp}}_{01},3\right)$$

where t is the day of observation; y_t_ is the death count of t^th^ day that follows the quasi-Poisson distribution; Strata_t_ is a categorical variable of year and calendar month to control the confounding long-term trend and seasonality, η is the corresponding vector of coefficients; Dow_t_ is the day of week of t^th^ day, and γ is the vector of coefficients; ns ( ) is the natural cubic spline. The terms mhumd_01_, mpressure_01_ and mtemp_01_ are the two days mean of relative humidity, barometric pressure and mean temperature, respectively.

We then added the PM_2.5_ terms in the primary adjusted model. Three cumulative lag structures, i.e., lag 0–1, lag 2–5 and lag 0–5 within the framework of an unconstrained distributed lag (UDL) model, were chosen to account for the lag effects of PM_2.5_ in accordance with previous studies [4,27,28]. This choice was viewed as a compromise between a priori and data-driven approaches for selecting the lag structure for different exposure/outcome combinations [4,27,28]. Multiple lag exposures were simultaneously added to the model and the cumulative effects were the sum of the single lag coefficients [29,30]. Moreover, the UDL approach can provide unbiased cumulative estimates for exposures of interest (i.e., PM_2.5_ in this case) the same as those from the polynomial distributed lag model [31,32]. The general formula of this UDL model is defined as the ”base model”, and can be expressed as:

(2) $$\log\left(y_{t}\right)=\alpha +{\eta \mathrm{Strata}}_{t}+{\gamma \mathrm{Dow}}_{t}+\mathrm{ns}\left({\mathrm{mhumd}}_{01},3\right)+\mathrm{ns}\left({\mathrm{mpressure}}_{01},3\right)+\mathrm{ns}\left({\mathrm{mtemp}}_{01},3\right)+\sum_{k=0}^{K}\beta_{k}{\mathrm{PM}}_{2.5t-k}$$

where PM_2.5t−k_ is concentration of PM_2.5_ k days before the day of death (t^th^ day); β_k_ indicates the corresponding coefficient; the other terms are the same as those in Equation (1). Given the already established linear relationship between particulate air pollutants and mortality [33], PM_2.5_ was modeled as a linear term in the base model.

In accordance with previous studies [4], we examined the dependency of daily deaths on PM_2.5_ of current day and of preceding days, up to the previous five days, firstly using a single lag model and then a third-degree polynomial distributed lag model.

Regarding the exploration of exposure-response relationships for PM_2.5_ with CVD, CBD and IHD mortality for the whole population, we fitted a natural cubic spline, rather than a linear function, with two equally spaced inner knots for the pollutant exposure within the framework of UDL model to simultaneously evaluate the potential nonlinear relations and the cumulative mortality effects of multiple-day exposure to PM_2.5_. The lowest value of PM_2.5_ during study period was used as the reference level. We also examined the mortality effects of PM_2.5_ and relevant exposure-response relationships stratified by age and gender. In particularly, the age group was classified as two groups: people aged <65 years old and ≥65 years old.

#### 2.3.3. Impacts of Extensive Adjustment of Temperature and Relative Humidity

Based on the models established in the previous Section 2.3.2, we then created a series of extensively adjusted models, which allow for the prolonged and nonlinear effects of temperature and relative humidity for different lag periods (days). In this case, terms of “cross-basis” function were introduced in the model to control the effect of temperature and relative humidity. Specially, a natural cubic spline with four degrees of freedom was used for both dimensions of temperature/humidity and lags over time (i.e., exposure days) [34]. Given the fact of no one temperature measure was superior to others, we only used the daily mean temperature to control its effects on mortality, which was more stable and had less missed records [35]. Candidate lags of 7, 14, 21, 28 and 40 days for temperature/humidity were adopted in extensively adjusted models. The selected lag periods with one week interval from minimum 7 days to 28 days were motivated by the evidence of an acute heat effect (hot temperature), which usually lasted for days within one week, and the prolonged cold effect (cold temperature), which could last for several weeks [12,34]. While the choice of 40 days as the maximum was used to make sure the effect of temperature was sufficiently controlled when exploring the impacts of different adjustments (i.e., controlling for different lag periods) of temperature on PM_2.5_ effects and corresponded exposure-response relationships. The general formula of the extensively adjusted model can be expressed as follows:

(3) $$\log\left(y_{t}\right)=\alpha +{\eta \mathrm{Strata}}_{t}+{\gamma \mathrm{Dow}}_{t}+s\left({\mathrm{temp}}_{t};\phi \right)+s\left({\mathrm{humd}}_{t};§\right)+\mathrm{ns}\left({\mathrm{mpressure}}_{01},3\right)+\sum_{k=0}^{K}\beta_{k}{\mathrm{PM}}_{2.5t-k}$$

where the s(temp_t_; ϕ) and s(humd_t_; §) were the defined cross-basis function for the temperature/humidity, with natural cubic spine as the basis for the dimension of temperature/humidity (temp_t_/humd_t_) and lags over time (ϕ/§). The other terms was as same as in Equation (2).

Through this, we assessed the impacts of different extents of adjustments for temperature and relative humidity on both PM_2.5_ effects and the corresponding exposure-response relationships for the whole population and subgroup population stratified by gender and age groups.

### 2.4. Sensitivity Analysis

We performed several sensitivity analyses to check the robustness of the primary results. In order to interpret the sensitivity results in briefly and more easily, we restricted the assessments to the whole population. First, we changed the length of time window to 21 and 30 days rather than the fixed calendar month, and we also replaced the combination factor terms of Strata (Strata_t_) and Dow (Dow_t_) by a factor term of three-way interaction among year, month and day of week. Second, we added the smoothed wind (for two days mean) into the base model and extensively adjusted models. Third, we estimated the associations between PM_2.5_ and cardiovascular mortality and refitted the corresponded exposure-response relationships based on extensively adjusted model with only adjusting for temperature. Fourth, we refitted the exposure-response relationships for PM_2.5_ with three types of cardiovascular disease mortality based on base model (i.e., adjusting for temperature/humidity for two days), changing the DF (from 2 to 6) of adopted natural cubic spline for the PM_2.5_ dimension. Fifth, we changed the DF (from 3 to 6) of the natural cubic spline for the dimension of temperature/humidity and lags in cross-basis terms in adjusted model. To illustrate briefly, the analyses were restricted to the lags of 21 days when assessing the PM_2.5_ effects. Finally, we restricted our analyses to districts within the monitoring radius of the U.S. embassy station. Specifically, the Quasi-AIC (QAIC, the AIC in quasi-Poisson regression methods) was used to assess the model fit when we changed the DF for the applied natural cubic splines.

The main results are expressed as percent increase in risk of death, with 95% confidence intervals (CIs), relative to 10 μg/m^3^ increment in PM_2.5_ unless specified otherwise. Spearman’s correlation was used to examine correlation relationships among the air pollutant and meteorological factors. The data preparation was via SAS, version 9.4 (Cary, NC, USA). We fitted all the models in R 3.2.2 (R Core Team, Vienna, Austria) using the dlnm [36] and mgcv [37] packages. Statistical significance was defined as *p*-value < 0.05 (two-sided).

## 3. Results

### 3.1. Descriptive Results

From 1 January 2008 to 31 December 2011, we recorded a total of 145,477 deaths from CVD, with males accounting for 54.84% (79,777) and females for 45.16% (65,700). Decedents with age ≥65 years old accounted for 80.71% (117,417) of the total CVD deaths. Table 1 summarizes the daily mortality, air pollutant and meteorological factors. On average, there were approximately 100 CVD deaths per day, including 46 from CBD and 45 from IHD. The average daily concentration of PM_2.5_ during present study period was 95.68 μg/m^3^, with highest value of 492.75 μg/m^3^ and lowest value of 5.83 μg/m^3^. During our study period, the average daily mean temperature and relative humidity were 13.21 °C and 50.86%, respectively.

As depicted in Supplementary Materials Table S3, the PM_2.5_ was positively correlated with daily mean temperature and relative humidity, with Spearman’s correlation coefficients of 0.17 and 0.58, respectively. In contrast, the barometric pressure and wind revealed reverse relations with PM_2.5_.

### 3.2. Associations and Exposure-Response Relationships between PM_2.5_ and Mortality in Whole Population and Subgroup Population from Base Models

Estimates from both single lag models and polynomial distributed lag models for the whole population clearly present the evidence of the immediate effects of the PM_2.5_ on CVD, CBD and IHD mortality, up to lag 1 (previous one day, see Supplementary Materials Figure S3). Similar evidence of immediate effects of PM_2.5_ on three outcome mortalities was also shown in males, females, people aged ≥65 years and <65 years, but estimates for the group with age <65 years were all non-significant (see Supplementary Materials Figures S4 and S5).

Table 2 reports the associations between PM_2.5_ and CVD, CBD and IHD mortalities for the three cumulative lags (lag 0–1, lag 2–5, lag 0–5). In general, the highest estimates for the three outcomes mortalities in whole population and subgroups were all observed at lag 0–1, indicating the immediate mortality effects of PM_2.5_. Meanwhile, estimates for the male and elderly (aged ≥ 65 years) populations were higher than that of females and the younger population, indicating males and the elderly population might be more susceptible to PM_2.5_ effects. Regarding the estimates across the three outcomes in the whole population, comparable magnitude associations were found for CVD and CBD mortality, while higher estimates were observed for IHD mortality compared with the former two outcomes, and this pattern was consistent in subgroups across gender and age group, with somewhat exception for younger population. Given these results and the results from both single lag and distribute lag models (see Supplementary Materials Figures S3–S5), we used lag 0–1 for the subsequently analysis of CVD, CBD and IHD mortality within the whole population and subgroup population.

**Figure 1:**
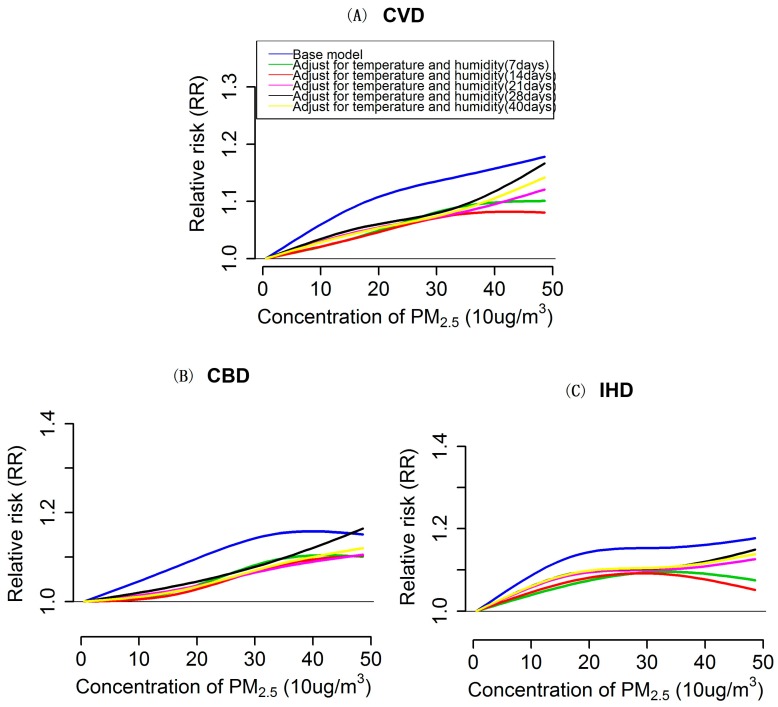
Exposure-response relationships for PM_2.5_ (lag 0–1) with cardiovascular (CVD), cerebrovascular (CBD), and ischemic heart disease (IHD) mortality for whole population, according to single pollutant UDL models. Values are estimated relative risk of mortality associated with PM_2.5_ relatives to lowest value (5.83 μg/m^3^). Base model(blue lines) adjusted effects of temperature and humidity only for two days ( modeled as two days moving average), whereas the lines with color of green, red, pink, black and yellow represent relationships from extensively adjusted models that with simultaneously adjusted cumulative effects of temperature and relative humidity for 7, 14, 21, 28 and 40 days, respectively.

**Figure 2:**
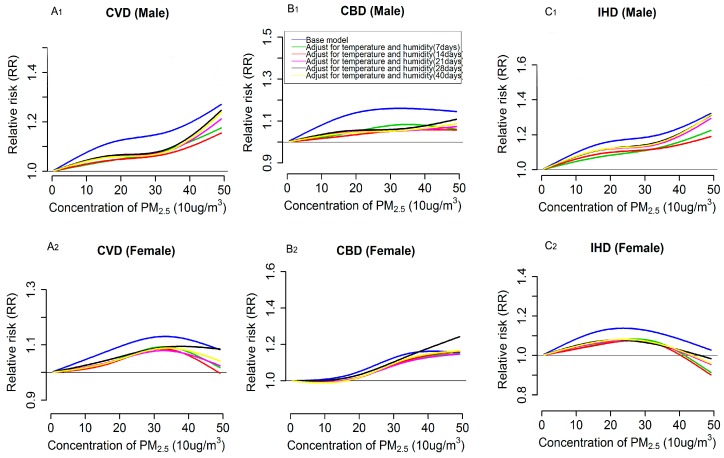
Exposure-response relationships for PM_2.5_ (lag 0–1) with cardiovascular (CVD, **A_1_**, **A_2_**), cerebrovascular (CBD, **B_1_**, **B_2_**), and ischemic heart disease (IHD, **C_1_**, **C_2_**) mortality by gender, according to single pollutant UDL models. Values are estimated relative risk of mortality associated with PM_2.5_ relatives to lowest value (5.83 μg/m^3^). Base model (blue lines) adjusted effects of temperature and humidity only for two days(modeled as two days mean), whereas the lines with color of green, red, pink, black and yellow represent relationships from extensively adjusted models that with simultaneously adjusted cumulative effects of temperature and relative humidity for 7, 14, 21, 28 and 40 days, respectively.

**Figure 3:**
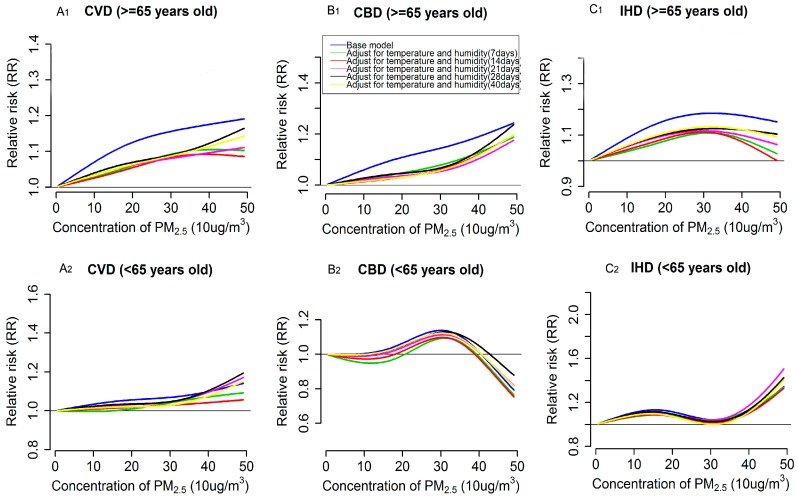
Exposure-response relationships for PM_2.5_ (lag 0–1) with cardiovascular (CVD, **A_1_**, **A_2_**), cerebrovascular (CBD, **B_1_**, **B_2_**), and ischemic heart disease (IHD, **C_1_**, **C_2_**) mortality by age group, according to single pollutant UDL models. Values are estimated relative risk of mortality associated with PM_2.5_ relatives to lowest value (5.83 μg/m^3^). Base model(blue lines) adjusted effects of temperature and humidity only for two days(represented as two days moving average), whereas the lines with color of green, red, pink, black and yellow represent relationship from extensively adjusted models that with simultaneously adjusted cumulative effects of temperature and relative humidity for 7, 14, 21, 28 and 40 days, respectively.

Besides the PM_2.5_ effects, we also modeled the exposure-response relationship for PM_2.5_ with cardiovascular mortality based on base models. Overall, for CVD and CBD mortality in the whole population, the exposure-response curves were approximately linear, consistent with the absence of a threshold, presenting as a generally monotonic increasing trend of relative risk of mortality along the PM_2.5_ exposure, but there exists a slight downward trend in the curve at the higher end of PM_2.5_ level for CBD mortality (see blue lines in Figure 1A,B). However, there was obvious plateau, in the range of approximately 200–400 μg/m^3^, in the relation for PM_2.5_ with IHD mortality (see blue lines in Figure 1C). When looking into the relevant exposure-response relations in subpopulation by gender and age group, we found that the curves of the relations for each outcome varied across subgroups. Specifically, the overall shape of the exposure-response relationships in susceptible population, i.e., those with higher estimates of PM_2.5_ effects (in this case, referred to males and the elderly), were generally consistent with that in the whole population but the slopes of the curves were steeper. The plateau in the curve for IHD mortality in the whole population was not observed in males, where a roughly linear relation was found, and the elderly population, where a bump was visually observed in the response curve at approximately 300 μg/m^3^ (see blue lines in Figure 2A_1_–C_1_ and Figure 3A_1_–C_1_). In contrast, for the exposure-response relationships of CVD and IHD mortality in female, evident bumps were observed in the curves at the range of 300–350 μg/m^3^ and 200–300 μg/m^3^, respectively (see blue lines in Figure 2A_2_–C_2_), but the exposure-responses curve for CBD in this group was roughly linear, consistent with the absence of a threshold. Additionally, the relation curves for the three outcomes in the population aged <65 years were poorly estimated, because of the fewer daily death counts contributing to the estimation (see blue lines in Figure 3A_2_–C_2_).

### 3.3. Impacts of Extensively Adjustment of Temperature and Humidity on PM_2.5_ Effects Estimation and Exposure-Response Relationships

Table 3 shows the estimated effects of PM_2.5_ on three types of cardiovascular disease mortalities after adjusting for temperature and humidity for different exposure days. Overall, the estimates for CVD, CBD and IHD mortalities in the whole population and subpopulations decreased dramatically after extensive adjustment for temperature and humidity for 7, 14, 21, 28 and 40 days when compared with the estimates from the base model (see Table 3). Notably, the extent of decrease in the estimates varied across subgroups, with a larger decrease occurring in the susceptible population (i.e., the elderly and males, in this case) compared with other groups. Similarly, the magnitudes of the exposure-responses relations were also dramatically attenuated when longer lag periods (more than one week) of temperature and humidity were included in the adjustments, and remarkable decreases were also observed in males and the elderly (see Figure 2 and Figure 3). Nevertheless, the associations between PM_2.5_ and the above three outcome mortalities generally held up, except for that in the < 65 years group, and the overall shape of the modeled exposure-response relations when extensively adjusting for temperature and humidity consistent matched those of the relations in the base models. However, the linear trend or the monotonic increasing trend of relative risk of mortality of the exposure-response curves for the three types of cardiovascular diseases in the whole population and subgroups became more apparent after the extensive adjustments (see Figure 1, Figure 2 and Figure 3). Meanwhile, the plateau in the exposure-response curve of IHD mortality for the whole population and the bumps in the relevant curves of CVD and IHD mortality for females became less evident when adjusting for temperature and humidity for more than 21 days (see Figure 1C and Figure 2A_2_–C_2_). Although, when compared with the results from the base model, there exist changes in the estimates of PM_2.5_ effects and the corresponding exposure-response relationships after the extensive adjustments of temperature and humidity, and we found that there might be no substantial difference among these extensive adjustments, which can be reflected in the similar estimates (see Table 3) and the highly overlapped exposure-response curves but somewhat dispersed trends in the curves’ tail (at approximately >400 μg/m^3^ ) (see Figure 1, Figure 2 and Figure 3) across the extensive adjustments.

### 3.4. Sensitivity Analysis Results

In general, as presented in Supplementary Materials Table S4, when we changed the choices of selected “strata”, additionally adjusted for wind, re-did the analysis only extensively controlling for temperature and focused on the restricted districts, the patterns of the changes in estimates of PM_2.5_ effects and the corresponding exposure-response curves for the whole population were similar to the primary findings in the whole population (see Supplementary Materials Figures S6 and S7). Varying the DF (from 2 to 6) of the natural cubic spline for PM_2.5_ in the base model did change the shape of the exposure-response curves, presenting as more wiggles appearing in the curves with the increase of DF, consequently, resulting in difficulty in providing scientific or biological explanations for the relations (see Supplementary Materials Figure S8). The performance of model fit, expressed by QAIC, favors the lower DF, that’s 2 and 3 (this DF was adopted in main analysis), where the curves of relationship highly overlapped but with some dispersion at higher levels of PM_2.5_ (approximately 400 μg/m^3^). Changing the DFs of the basis function for the both dimension of cross-basis terms of temperature/humidity did not substantially alter the estimates of PM_2.5_ effects, but the QAIC indicates a better model fit for the adopted combined DF in the present main analysis (see Supplementary Materials Table S5).

## 4. Discussion

In our study, we first explored the effects of PM_2.5_ on CVD, CBD and IHD mortalities and modeled the relevant exposure-response relationships based on base models, where the effects of temperature and humidity were controlled for two days in accordance to the approach used in previous multicity studies, using the mortality data for 2008 through 2011 in Beijing. Then we constructed a series of models extensively adjusted for temperature and humidity. The comparison of the PM_2.5_ effects and corresponded exposure-response relations across the models that with different adjustments (including that in base model) for temperature and humidity shows that the including of longer lag period of temperature and humidity for adjustments can pose changes in PM_2.5_ effects and relevant exposure-response relations, and these changes varied across subpopulation group by gender and age.

The control for potential confounders when evaluating the acute effects of PM_2.5_ and other air pollutants on mortality in time series studies or case crossover studies mainly focuses on controlling unmeasured confounding factors, especially for the long-term and seasonal trends of time [8,10,38], while less attention was paid to the concerns whether the effects of temperature and other meteorological factors have been well modeled [14]. In fact, if the long-term trend and seasonality were well controlled, reflecting the fact there would be no apparent long-term patterns in the distribution of residuals of the fitted model, and any additional control for the other unmeasured factors such as “holiday” would not substantially improve the model fit [39]. In this case, further controlling for the measured and time-varying factors plays a key role in obtaining the valid effects of air pollutants. Of those measured factors, temperature and humidity, especially temperature, would be the most important factors that should be allowed for. As a time-varying measured factor, temperature is not only associated with the daily cardiovascular mortality but also related to the level of air pollutants, consequently, the temperature would be the most important confounder of air pollutant effects [33,39]. Consistent evidences have shown that the effects of temperature on mortality are higher than those of air pollutants (including PM_2.5_), thus inadequately controlling for temperature would have a great impact on the associations between PM_2.5_ and cardiovascular mortality.

In the present investigation, our results illustrated the estimated effects of PM_2.5_ and magnitude of the relevant exposure-response relationships based on base models markedly decreased after extensively adjusting for the temperature and relative humidity for 7, 14, 21, 28 and 40 days, indicating that allowing for the effects of temperature and humidity only for days within one week inevitably results in overestimated PM_2.5_ effects. Meanwhile, we also observed that the estimates of PM_2.5_ effects were generally significant in the whole population and most subpopulations, in particular for males, females, and the elderly group, even after adjusting for temperature and humidity for more than two weeks, which provides additional evidence of the observed significant and positive association between PM_2.5_ and the cardiovascular mortality reported in previous studies (see listed study in Supplementary Materials Table S2) that only controlled for temperature for a few days was not an artifact of the inadequately modeled temperature. A similar conclusion can be drawn from Welty and Zeger’s work [14], where they conducted a series of sensitivity analyses, where two versions of flexible distributed lag models were used to model the effect of temperature, to check whether the reported acute effects of particulate matter (PM_10_) in the National Morbidity, Mortality , and Air pollution Study (NMMAPS) were the result of inadequate control for the weather and season. They found that the original results in NMMAPS were robust to the adjustments of temperature, but they only focused on PM_10_ at single lag for total/natural causes deaths and the exposure days for the temperature were limited to two weeks. Studies have shown the consistent cumulative and nonlinear effects of temperature on mortality, in which the effects, especially for cold temperature, can last for approximately one month or more [12], and researchers also recognized the importance of adequate control for temperature and other weather related factors [40]. We only found one study that made extensive adjustments for the cumulative effects of meteorological factors for the previous 27 days for the evaluation of PM_2.5_ effects, in which the results were consistent with ours and that study was also conducted in Beijing but only focused on all natural cause mortality [41]. We didn’t conduct extensive controlling for the wind and barometric pressure since there is insufficient evidence that these two factors are confounders of the PM_2.5_ effects.

Furthermore, we found that the changes in PM_2.5_ effects and corresponding exposure-response relations before (i.e., results in the base model) and after extensive adjustments for temperature and humidity for different lag periods (days) varied across subgroups, with marked decreases in estimated effects of PM_2.5_ and magnitude of exposure-response relations observed in males and the elderly population, for which higher estimates of PM_2.5_ effects were found (see Table 3). This variation in changes might because those susceptible populations were also susceptible to the mortality effects of temperature, consequently, the control for temperature could dramatically affect the effects of PM_2.5_. Previous studies have concluded that the temperature can pose greater mortality effects on males and elderly people than other groups [42]. Besides, when we looked into the shape of the exposure-response curves for each type of cardiovascular mortality in the different subgroups, we found that the curves in males and the elderly were, generally, roughly linear without evidence of thresholds, whereas there exists smooth bumps with a downward trend at higher level of PM_2.5_ (at least up to 200 μg/m^3^) for females, especially for CVD and IHD mortality, which might partly reflect the previously observed saturation phenomenon in the particle exposure response [43,44,45], where the cardiovascular risk would not sustain an increase over the whole increase in particle exposure because the underlying biochemical and cellular processes involved may become statured with small doses of toxic components [45]. Notably, this phenomenon was initially proposed in the evaluation of the pathophysiology of cigarette smoke exposure and cardiovascular disease [45], and evidence from studies of air pollution-related cardiovascular mortality, especially for those evaluating the acute effects of PM_2.5_ in different susceptible populations, are still insufficient. Therefore, further studies with a large range of PM_2.5_ exposures and sufficient sample sizes are deeply needed to confirm the existence of the saturation phenomenon and to better quantify the shape of exposure-response curves in subgroups with varied susceptibility. Certainly, the probability of uncertainly from statistical modeling, such as different DFs for the adopted splines and the different types of smoothers, cannot be completely ruled out since varying the DFs and smoothers might also affect the shapes of exposure-response relations and we only performed the sensitivity analysis by changing DF for the natural cubic splines rather than switching to another smoother such as penalized smoothing splines. Additionally, the downward trend after those bumps might be attributed to small daily death counts in days with higher levels of PM_2.5_ [13], which also might be the major reason for the observed dispersion in the tails of the exposure-response curves for the whole population and subgroups. In contrast, the limited daily death counts for the <65 years age group impeded us from drawing credible conclusions about this group. The observed roughly linear relations in the susceptible populations (males, aged ≥65 years group) strongly indicates that effective measures should be taken to protect them from the hazardous effects of PM_2.5_.

Although we have performed five extensive adjustments of temperature and humidity for a series of longer lag periods of 7, 14, 21, 28 and 40 days, we found that estimates of PM_2.5_ effects were generally similar and the 95% CIs were overlapped. Meanwhile the exposure-response curves were also highly overlapped, except for the dispersion in the tails of curves, across these five adjustments. These finding suggested that further adjusting for the temperature and humidity for more than one week did not substantially alter the estimates of PM_2.5_ effects and the magnitude of the exposure-response relations. This might be because the strongest effects of temperature, especially for heat effects, were commonly found on current day and the past few days [26,34,46], and extending the lags to longer periods such as more than one week might result in less evident changes in estimates but did result in the changes in the significance of these estimates. However, we have to note that adjusting for temperature for a few days within one week might inadequately capture the overall cumulative effects of temperature. Consistent evidence has shown that the effects of cold temperature last for several weeks, whereas the heat effects last for a few days within a week [12,34]. Combining our results from the base model with the aforementioned evidence, it would be better to control for the effect of temperature (and humidity) for at least for 7 days in order to adequately capture the heat effects and somewhat the cold effects, but extending the lags up to 21 days might sufficiently control for the overall cumulative temperature effects, including the prolonged cold effects, and the lag period of 21 days is also commonly adopted in studies of temperature-related mortality [12,26,34]. In addition, though the changes in the size of effects were small among the different extensive adjustments, we found that the observed roughly linear trend in curves in the whole population and subgroups became more evident and the plateaus or bumps seem less apparent after the extensive adjustments for temperature, especially for 21 days, which reminds us to interpret with caution the previously reported nonlinearity of the threshold relation for PM_10_ with mortality in several Chinese cities [47], where the temperature were only modeled for days within one week, for which might be artifacts of inadequately controlled temperature and humidity. However, our results were based on a single city, so cautions still should be taken when the findings of the present investigation are generalized to other sites. Thus, multicity approaches are warranted in the future to confirm our results. Nevertheless, the present exploration of the impacts of controlling for temperature and humidity for different days on both effects of PM_2.5_ on cardiovascular event mortalities and the relevant exposure-response relationships can offer an empirical guide for obtaining relatively accurate deleterious effect data for other pollutants in future studies.

Several limitations in the present study should be acknowledged. First, the main results were drawn from observational data, and simulation analysis related to this topic were not performed, which might reduce the scientific soundness of our findings, and further studies should be conducted to address this issue. Secondly, we only obtained PM_2.5_ data from the U.S. embassy, which is located in Chaoyang District, a central district of Beijing, thus the PM_2.5_ levels cannot represent the whole city level in Beijing. Thirdly, we did not consider the personal information of daily activity patterns, indoor exposures to PM_2.5_, and chronic disease-related risk factors, which inevitably hinders estimating the impacts of those aspects on the mortality effects of PM_2.5_. The present investigation was limited to PM_2.5_, and other pollutants were not considered, but the present adopted approach can still be applied in the presence of co-pollutants. Fourthly, the imputed PM_2.5_ values might increase the uncertainly of our estimates, though the adopted method fitted well the predicted values and observed values. Finally, we assumed the effects of PM_2.5_ were constant across years through the present study period (from 2008 to 2011), but studies have stated that the annual levels of PM_2.5_ in China were slightly reduced from 2008 to 2013 [48], which raises the issue whether the effects of PM_2.5_ would decrease across years or different periods. We only used the data of four years, which might be too short to detect the this hypothesis_。_.

## 5. Conclusions

In summary, the present study provided evidence that the residual confounding of temperature and other meteorological factors was not responsible for the observed effects of PM_2.5_ on cardiovascular event mortalities (i.e., CVD, CBD and IHD). The male and elderly population were more susceptible to PM_2.5_ effects, with higher estimates observed in those subgroups. The inadequately controlled temperature and humidity could lead to overestimated PM_2.5_ effects. The changes in estimated effects of PM_2.5_ and the magnitude of the corresponding exposure-response curves were similar across the different extensive adjustments of temperature and humidity, but those changes varied across subgroups, with markedly decreased in males and the elderly. A multicity approach would be more important to obtain the generalized findings, offering more convincing findings to support the national air quality control and public health protection.

## Figures and Tables

**Table 1:** Descriptive statistics of daily death counts, ambient air pollutants and meteorological factors in Beijing, China (2008–2011).

Factor	Mean ± SD	Percentiles				
		Min	P_25_	P_50_	P_75_	Max
**Outcomes**						
CVD	99.57 ± 20.36	54	85	97	113	173
CBD	46.25 ± 10.32	22	39	45	53	82
IHD	44.87 ± 11.18	20	37	44	52	92
**Environmental Data**						
PM_2.5_ (μg/m^3^)	95.68 ± 70.83	5.83	41.79	80.09	127.92	492.75
Temperature (°C)	13.21 ± 11.34	−12.5	2.2	14.9	24.0	34.5
Barometric Pressure (kPa)	101.24 ± 1.03	98.97	100.41	101.18	102.05	103.93
Relative Humidity (%)	50.86 ± 19.97	9	34	52	67	95
Wind (m/s)	2.23 ± 0.93	0.5	1.6	2.1	2.7	6.4

Abbreviations: SD (standard deviation), Px (xth percentiles), Min (minimum), Max (Maximum); CVD: cardiovascular disease; CBD: cerebrovascular disease; IHD: ischemic heart disease.

**Table 2:** Estimated percent increase (95% CI) in risk of death associated with per 10 μg/m^3^ increase in PM_2.5_ by death causes in whole and subgroup population in Beijing, China (2008–2011) *.

Mortality	Lag Days	Whole Population	Gender		Age Group	
			Male	Female	≥65	<65
CVD						
	Lag 0–1	0.42 (0.28, 0.56)	0.47 (0.29, 0.64)	0.36 (0.16, 0.56)	0.46 (0.31, 0.62)	0.23 (−0.06, 0.52)
	Lag 2–5	−0.15 (−0.29, −0.02)	−0.10 (−0.27, 0.07)	−0.22 (−0.41, −0.03)	−0.14 (−0.29, 0.01)	−0.20 (−0.47, 0.08)
	Lag 0–5	0.24 (0.05, 0.43)	0.34 (0.10, 0.58)	0.12 (−0.15, 0.39)	0.30 (0.08, 0.51)	−0.01 (−0.41, 0.39)
CBD						
	Lag 0–1	0.42 (0.23, 0.62)	0.46 (0.21, 0.70)	0.38 (0.10, 0.66)	0.45 (0.24, 0.66)	0.31 (−0.12, 0.75)
	Lag 2–5	−0.16 (−0.34, 0.02)	−0.03 (−0.26, 0.20)	−0.33 (−0.60, −0.06)	−0.14 (−0.35, 0.06)	−0.23 (−0.64, 0.18)
	Lag 0–5	0.23 (−0.03, 0.50)	0.39 (0.05, 0.72)	0.03 (−0.36, 0.42)	0.29 (0.00, 0.58)	−0.02 (−0.62, 0.58)
IHD						
	Lag 0–1	0.47 (0.26, 0.67)	0.58 (0.31, 0.84)	0.34 (0.05, 0.62)	0.52 (0.29, 0.74)	0.23 (−0.21, 0.66)
	Lag 2–5	−0.23 (−0.43, −0.04)	−0.26 (−0.51, −0.01)	−0.20 (−0.47, 0.07)	−0.21 (−0.42, 0.01)	−0.35 (−0.77, 0.06)
	Lag 0–5	0.22 (−0.06, 0.50)	0.28 (−0.08, 0.64)	0.16 (−0.24, 0.55)	0.28 (−0.02, 0.59)	−0.07 (−0.67, 0.53)

* Estimated effects of PM_2.5_ were based on the single pollutant UDL models. Abbreviations: CVD: cardiovascular disease; CBD: cerebrovascular disease; IHD: ischemic heart disease.

**Table 3:** Percent increase (95% CI) in risk of death associated with per 10 ug/m^3^ increase in PM_2.5_ by gender, age group and death causes based on models with different extensive adjustments for cumulative effects of both temperature and relative humidity *.

Outcome	Population	Base Model ^a^	Extensive Adjusted Model ^b^				
			7 Days	14 Days	21 Days	28 Days	40 Days
CVD	Whole population	0.42 (0.28, 0.56)	0.25 (0.11, 0.40)	0.22 (0.07, 0.37)	0.24 (0.09, 0.38)	0.27 (0.13, 0.42)	0.25 (0.11, 0.39)
	Male	0.47 (0.29, 0.64)	0.26 (0.07, 0.44)	0.21 (0.02, 0.40)	0.25 (0.06, 0.44)	0.28 (0.10, 0.47)	0.26 (0.08, 0.43)
	Female	0.36 (0.16, 0.56)	0.25 (0.04, 0.46)	0.22 (0.01, 0.44)	0.21 (0.00, 0.43)	0.26 (0.05, 0.46)	0.24 (0.04, 0.44)
	≥65	0.46 (0.31, 0.62)	0.28 (0.11, 0.44)	0.25 (0.08, 0.41)	0.25 (0.08, 0.41)	0.29 (0.13, 0.45)	0.28 (0.12, 0.43)
	<65	0.14 (−0.16, 0.43)	0.15 (−0.17, 0.47)	0.10 (−0.22, 0.42)	0.18 (−0.14, 0.50)	0.19 (−0.13, 0.50)	0.13 (−0.18, 0.43)
CBD							
	Whole population	0.42 (0.23, 0.62)	0.26 (0.05, 0.47)	0.22 (0.01, 0.43)	0.21 (0.00, 0.42)	0.27 (0.06, 0.47)	0.23 (0.03, 0.42)
	Male	0.46 (0.21, 0.70)	0.22 (−0.04, 0.49)	0.15 (−0.11, 0.42)	0.16 (−0.11, 0.43)	0.19 (−0.07, 0.45)	0.17 (−0.08, 0.42)
	Female	0.38 (0.10, 0.66)	0.31 (0.00, 0.61)	0.31 (0.00, 0.62)	0.28 (−0.03, 0.60)	0.35 (0.05, 0.67)	0.30 (0.01, 0.60)
	≥65	0.45 (0.24, 0.66)	0.28 (0.05, 0.51)	0.23 (0.01, 0.46)	0.21 (−0.02, 0.43)	0.26 (0.04, 0.48)	0.22 (0.01, 0.43)
	<65	0.31 (−0.12, 0.75)	0.17 (−0.30, 0.65)	0.18 (−0.30, 0.67)	0.25 (−0.23, 0.74)	0.31 (−0.16, 0.78)	0.26 (−0.19, 0.72)
IHD							
	Whole population	0.47 (0.26, 0.67)	0.27 (0.05, 0.49)	0.25 (0.03, 0.47)	0.30 (0.08, 0.53)	0.33 (0.11, 0.54)	0.33 (0.12, 0.54)
	Male	0.58 (0.31, 0.84)	0.36 (0.05, 0.62)	0.35 (0.06, 0.64)	0.43 (0.14, 0.72)	0.47 (0.18, 0.75)	0.46 (0.19, 0.73)
	Female	0.34 (0.05, 0.62)	0.16 (−0.14, 0.47)	0.14 (−0.18, 0.45)	0.15 (−0.16, 0.47)	0.16 (−0.14, 0.47)	0.18 (−0.12, 0.47)
	≥65	0.52 (0.29, 0.74)	0.28 (0.04, 0.52)	0.28 (0.03, 0.52)	0.31 (0.07, 0.56)	0.35 (0.12, 0.59)	0.37 (0.14, 0.60)
	<65	0.23 (−0.21, 0.66)	0.23 (−0.25, 0.71)	0.14 (−0.35, 0.62)	0.25 (−0.24, 0.74)	0.21 (−0.27, 0.69)	0.15 (−0.31, 0.61)

Abbreviation: CVD: cardiovascular disease; CBD: cerebrovascular disease; IHD: ischemic heart disease.* Estimates were based on single pollutant UDL model at lag 0–1. ^a^ Model adjusted for effects of temperature and relative humidity simultaneously only for two days (modeled as two days moving average). ^b^ Model adjusted for cumulative effects of temperature and relative humidity simultaneously for 7, 14, 21, 28 and 40 days, respectively.

## Supplementary Materials

Supplemental materials are available at www.mdpi.com/1660-4601/13/11/1082/s1. Figure S1: Location of the U.S. embassy and the 12 national air quality monitoring (AQM) stations. Figure S2: Scatterplot of model fitting and validation for the imputation of PM_2.5_ (2011–2012). Figure S3: Lagging pattern of associations between PM_2.5_ and cardiovascular (CVD), cerebrovascular (CBD) and ischemic heart disease (IHD) mortality for whole population. Figure S4: Lagging pattern of associations between PM_2.5_ and cardiovascular (CVD), cerebrovascular (CBD) and ischemic heart disease (IHD) mortality by gender. Figure S5: Lagging pattern of associations between PM_2.5_ and cardiovascular (CVD), cerebrovascular (CBD) and ischemic heart disease (IHD) mortality by age group. Figure S6: Exposure-response relationships for PM_2.5_ (lag 0–1) with cardiovascular (CVD), cerebrovascular (CBD), and ischemic heart disease (IHD) mortality from extensively adjusted models for the whole population, according to different window lengths of the fixed-disjoint strata that used to control the long-term trend and seasonality. Figure S7: Exposure-response relationships for PM_2.5_ (lag 0–1) with cardiovascular (CVD), cerebrovascular (CBD), and ischemic heart disease (IHD) mortality from extensively adjusted models for the whole population, according different modelling strategies. Figure S8: Cumulative exposure-response relationship between PM_2.5_ and cardiovascular (CVD), cerebrovascular (CBD) and ischemic heart disease (IHD) mortality from the base model with different DF for a natural cubic spline in the PM_2.5_ exposures space. Table S1: Missing proportion of the collected PM_2.5_ data in the present study. Table S2: Comparison of the mortality effect of PM_2.5_ on cardiovascular events in studies in developed countries and China (estimates of PM_2.5_ were showed as percent increase in risk and corresponded 95% CI per 10 μg/m^3^ increase in concentration). Table S3: Spearman correlation coefficients between ambient pollutants and meteorological factors. Table S4: Sensitivity analysis for the associations between three type of cardiovascular disease mortality and PM_2.5_ according to different model choices. Table S5: Sensitivity analysis of associations between PM_2.5_ and mortality after controlling for temperature /humidity for 21 days with different DF for temperature/humidity (from 3 to 6) space and lag-space (from 3 to 6) selected in the cross-basis function.
