# Supplementary material for: Ambient Fine Particulate Matter Exposure and Risk of Cardiovascular Mortality: Adjustment of the Meteorological Factors
Source: Int J Environ Res Public Health. 2016 Nov 4;13(11):1082. doi: 10.3390/ijerph13111082 (PMC5129292; doi:10.3390/ijerph13111082)
Supplement: Supplementary file 1 [file ijerph-13-01082-s001.pdf]

Supplementary Materials: Ambient Fine Particulate Matter Exposure and Risk of Cardiovascular Mortality: Adjustment of the Meteorological Factors

Kai Luo, Wenjing Li, Ruiming Zhang, Runkui Li, QunXu and Yang Cao

Interpolation of missing values for daily $PM_{2.5}$ data

For the present study, data of the daily concentrations of $PM_{2.5}$ between 1 January 2008 and 31 December 2015 was retrieved from the U.S. embassy air pollution monitoring station (data available from the official website: http://www.stateair.net/web/post/1/1.html), which is located in the Chaoyang District of Beijing. Figure S1 shows the location of the China national air quality monitoring stations and the U.S. embassy monitoring station during the study period**.**

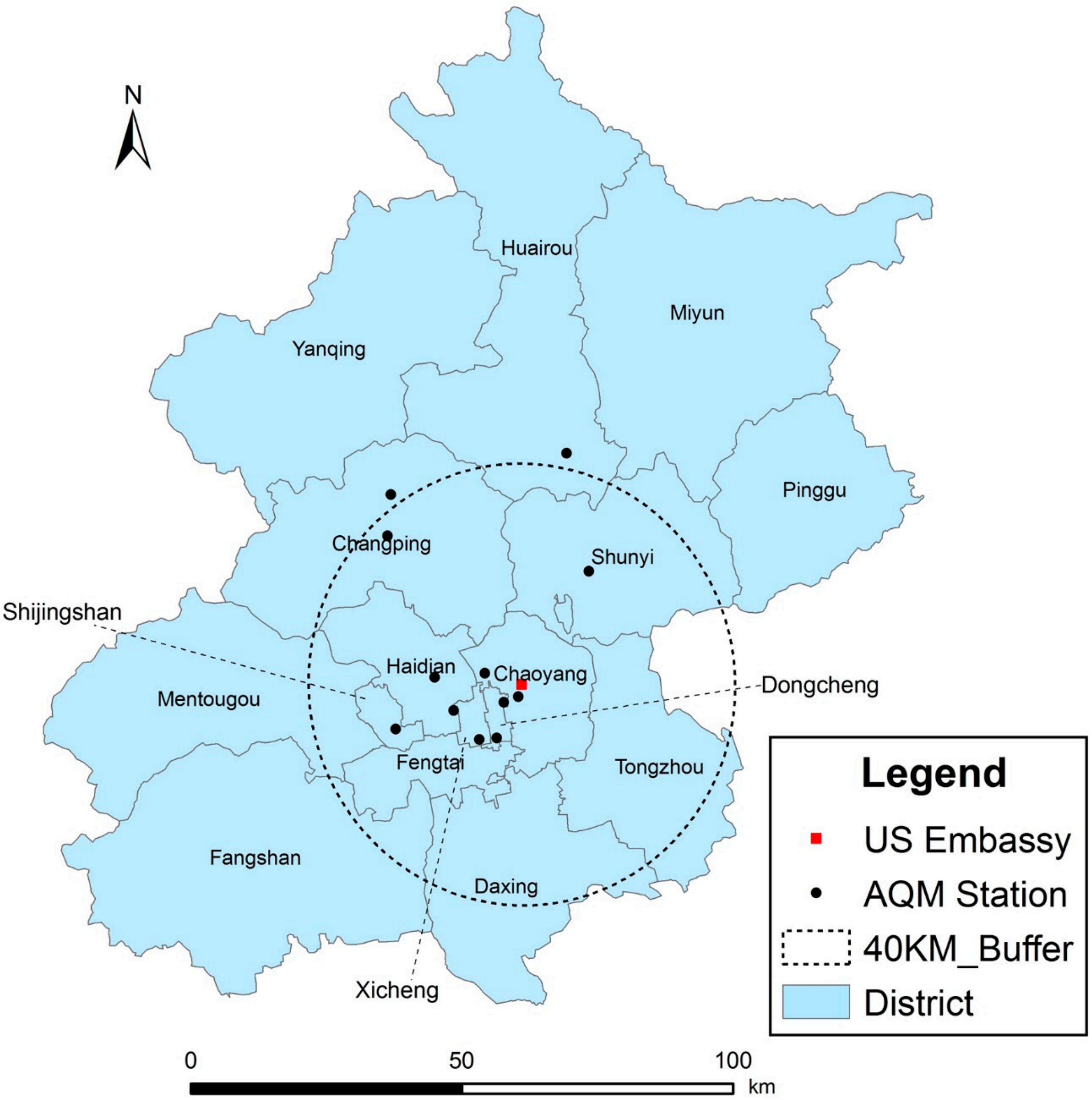

Figure S1. Location of the U.S. embassy and the 12 national air quality monitoring (AQM) stations (this map was created with ArcGIS Desktop version 9.3, ESRI, Redlands, California, USA, URL:http://www.esri.com/).

However, there were missing values for the obtained air pollution data, see Table S1. Therefore, we established a series of air pollutant predictive models to interpolate the missing values for the specific days rather than simply excluding them, which could reduce the statistical power of our present study. If the dataset with missing daily air pollutant values is less than 5% of the total, we regard the dataset as a complete dataset which will not introduce any bias in the estimation of the effects of air pollutants [1].

Table S1. Missing proportion of the collected $PM_{2.5}$ data in the present study [a].

Year	$PM_{2.5}$	
**(Full Days)**	**Missing Days**	**Missing Percent (%)**
2008 (366)	154	42.08
2009 (365)	64	17.53
2010 (365)	14	3.83
2011 (365)	11	3.01
2012 (366)	11	3.01
2013 (365)	0	0
2014 (365)	0	0
2015 (365)	0	0

[a] Daily concentrations of $PM_{2.5}$ were retrieved from station in U.S embassy in Beijing.

In brief, we divided the whole dataset into three parts. Two of the three parts were the complete dataset that will be used to build the predictive models and to perform the validation evaluation of those models. The rest was the dataset with missing values (more than 5%), and missing values were interpolated using the proposed predictive models. The details of this process are as follows:

Based on the potential relationship between air pollutants with meteorological factors, a series of generalized additive models (GAM) [2] were constructed with the logarithm of air pollutants as the dependent variables and the meteorological factors as independent variables. The associations between air pollutants and meteorological factors were modelled with penalized smoothing splines. In order to remove the temporal trends in the daily air pollutant and meteorological factors series when we built a predictive model, we included the dummy indicators of month and dow rather than simply modelling the time term with penalized smoothing function, which could result in overestimation and extreme values for the interpolated values. The general formula of GAM is given as

$$E(y_i) = \beta_0 + \text{factor}(\text{month}) + \text{factor}(\text{dow}) + f_1(\text{temp}_i) + f_2(\text{humd}_i) + f_3(\text{suntime}_i) + f_4(\text{wind}_i) + f_5(\text{pressure}_i) \quad (1)$$

Here, y_i is the ith day air pollutant (logarithm); month is the dummy variable for the month of year; f() are the penalized smoothing function; dow is the dummy variable for the day of week; the tempi represents the ith day temperature metrics includes mean temperature, maximum temperature and minimum temperature, measured as °C; the humd_i represents the ith day relative humidity, measured as %; the suntime_i is the ith day sun time measured as hour; the wind_i represent the ith day wind metrics includes the mean wind, maximum wind and minimum wind, all measured as m/s and the link function is identity. For the temperature or wind metrics, only one of the three metrics was added to the model at one time. An automatic choice was adopted to determine the dimension of the penalized smoothing spline based on generalized cross validation (GCV). The best predictive models were selected according to the magnitude of R-square during the validation of the predictive models.

For the $PM_{2.5}$, the dataset for 1 January 2013 through 31 December 2014 was used to create the predictive model. The dataset from 1 January 2011 to 31 December 2012 was used to test the validation of the created predictive models. The model with the best goodness of fit and highest R-square value was chosen as the best candidate predictive model, which will be used to interpolate the missing value for the dataset from 1 January 2008 to 31 December 2010. In addition, we also used the selected model to interpolate the missing values for 2011, because 3.01% of daily values of $PM_{2.5}$ in this year were missing. For this section, the R-square of the final selected model was 0.5367 with Pearson correlation coefficient of 0.7331, and the scatterplots of observed values against predicted values in 2011–2012 were displayed in the Figure S2. In summary, the predicted values from the final predictive

models matched well with the observed values for the $PM_{2.5}$.

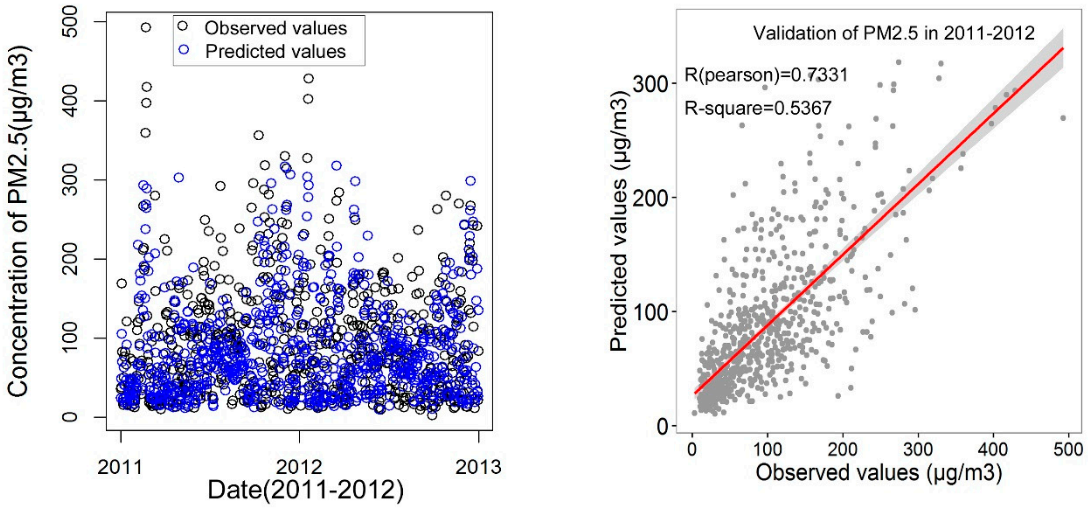

Figure S2. Scatterplot of model fitting and validation for the imputation of $PM_{2.5}$ (2011–2012).

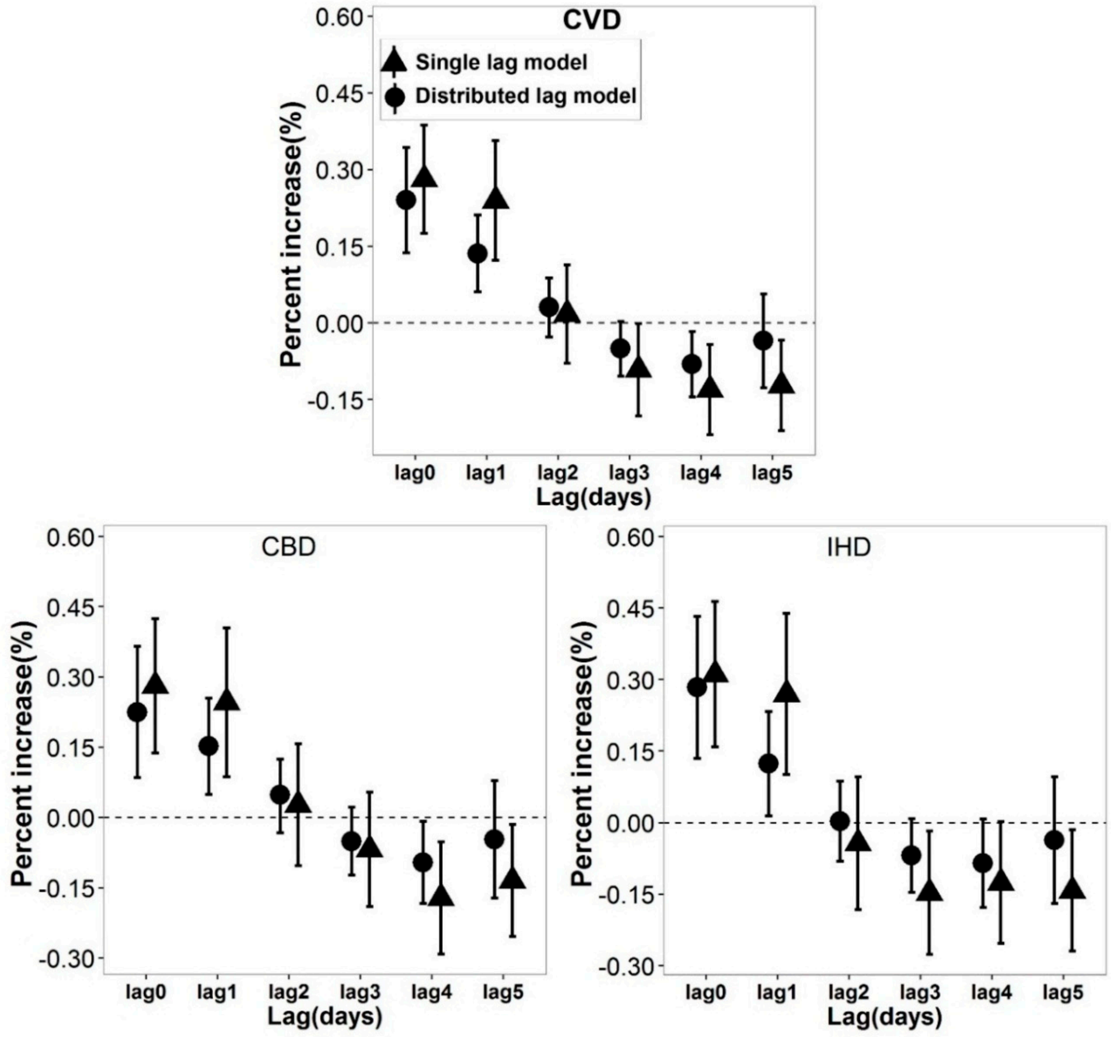

Figure S3. Lagging pattern of associations between $PM_{2.5}$ and cardiovascular (CVD), cerebrovascular (CBD) and ischemic heart disease (IHD) mortality for whole population. Specifically, a third-degree polynomial constraint was applied for distributed lag models for lags from 0 to 5 days.

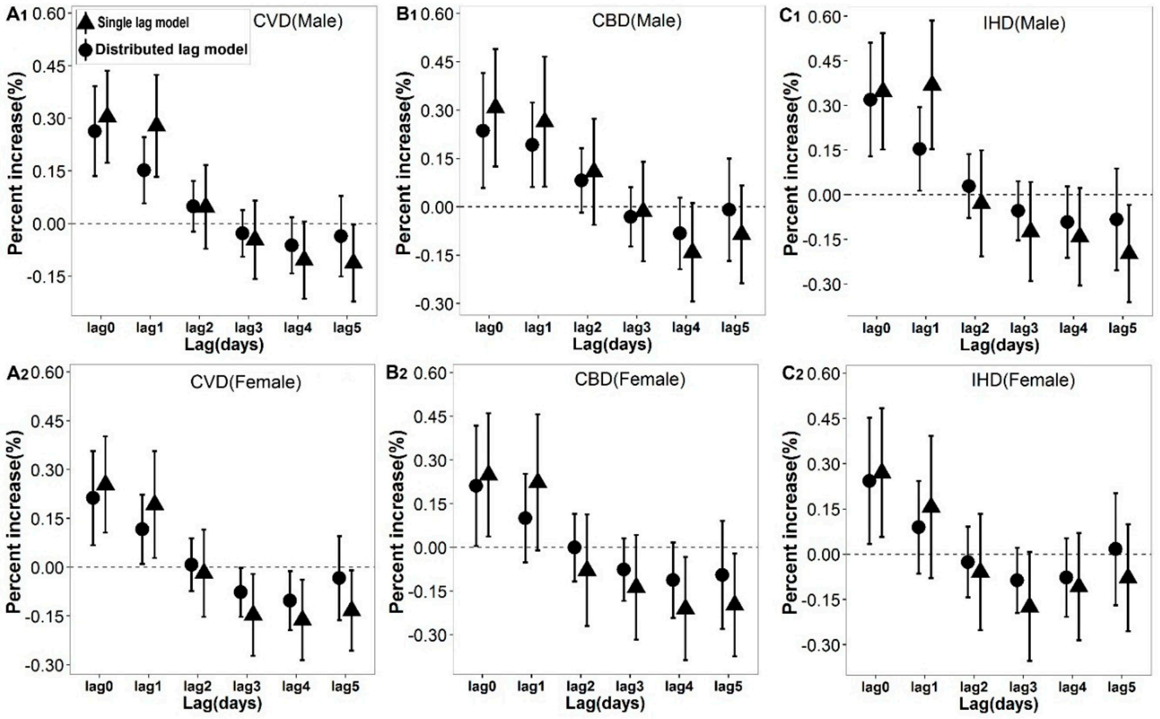

Figure S4. Lagging pattern of associations between $PM_{2.5}$ and cardiovascular (CVD), cerebrovascular (CBD) and ischemic heart disease (IHD) mortality by gender. Specifically, a third-degree polynomial constraint was applied in distributed lag models for lags from 0 to 5 days.

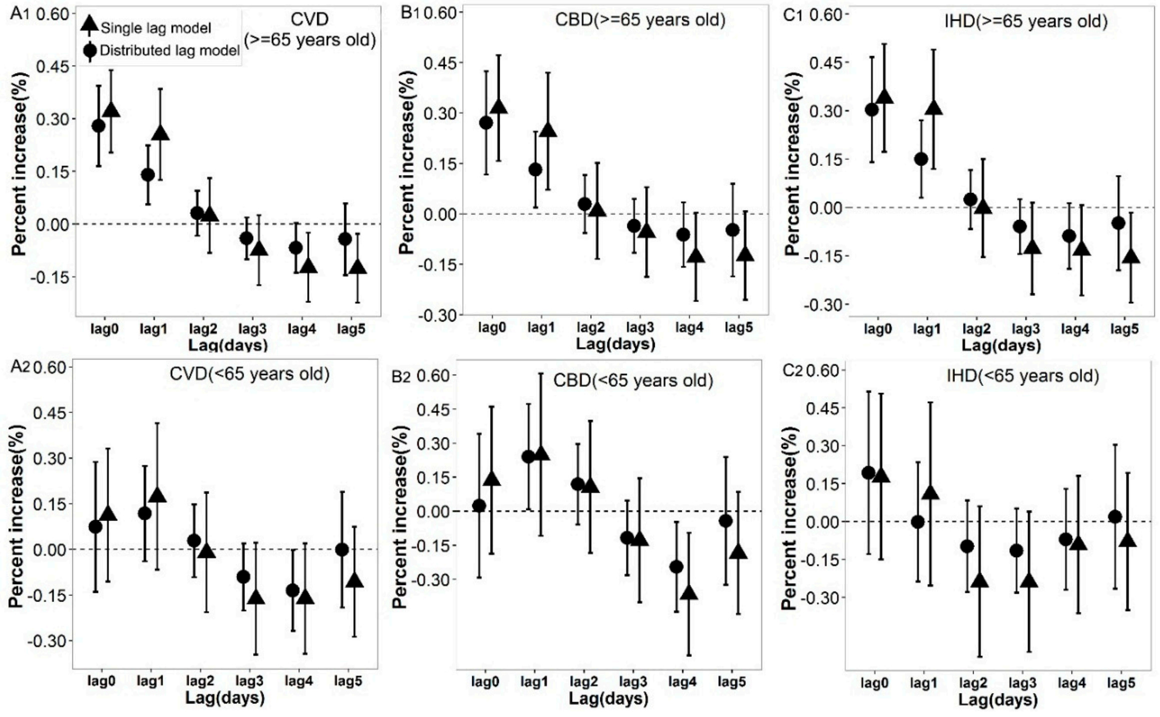

Figure S5. Lagging pattern of associations between $PM_{2.5}$ and cardiovascular (CVD), cerebrovascular (CBD) and ischemic heart disease (IHD) mortality by age group. Specifically, a third-degree polynomial constraint was applied in distributed lag models for lags from 0 to 5 days.

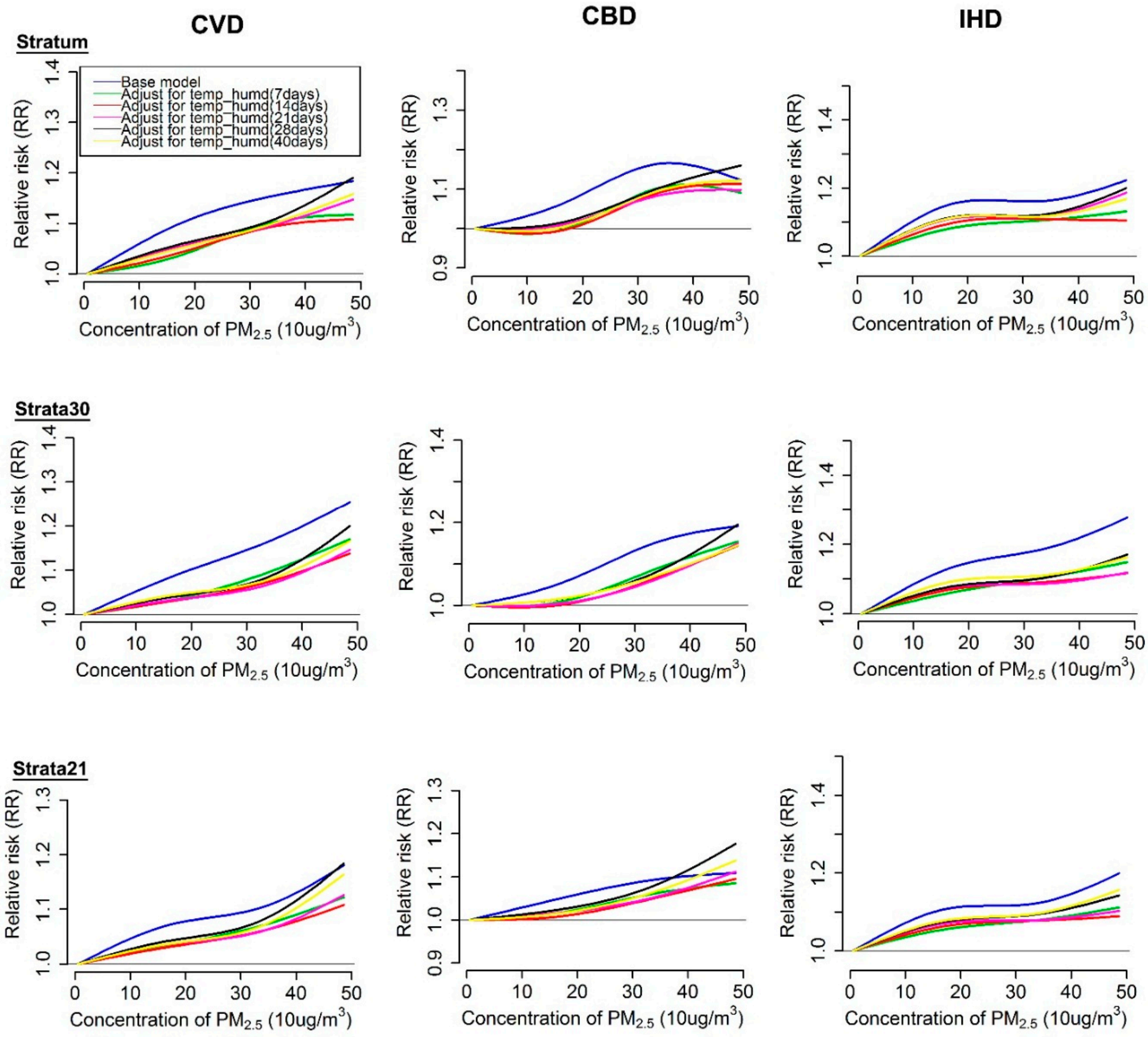

Figure S6. Exposure-response relationships for $PM_{2.5}$ (lag 0–1) with cardiovascular (CVD), cerebrovascular (CBD), and ischemic heart disease (IHD) mortality from extensively adjusted models for the whole population, according to different window lengths of the fixed-disjoint strata that used to control the long-term trend and seasonality. "Strata-21 days" and "strata-30 days" represent fixed and disjoined strata of 21 and 30 days in the same year were used to select the control days, while the stratum present the control days were selected as the same day of the week in the same month and year as the case period. Values are estimated relative risk of mortality associated with $PM_{2.5}$ relatives to lowest value (5.83 $\mu g/m^3$). Base model (blue lines) adjusted effects of temperature and humidity only for two days, whereas the green, red, pink, black and yellow lines represent relationships from models with extensively adjusted cumulative effects of temperature and humidity for 7, 14, 21, 28 and 40 days, respectively.

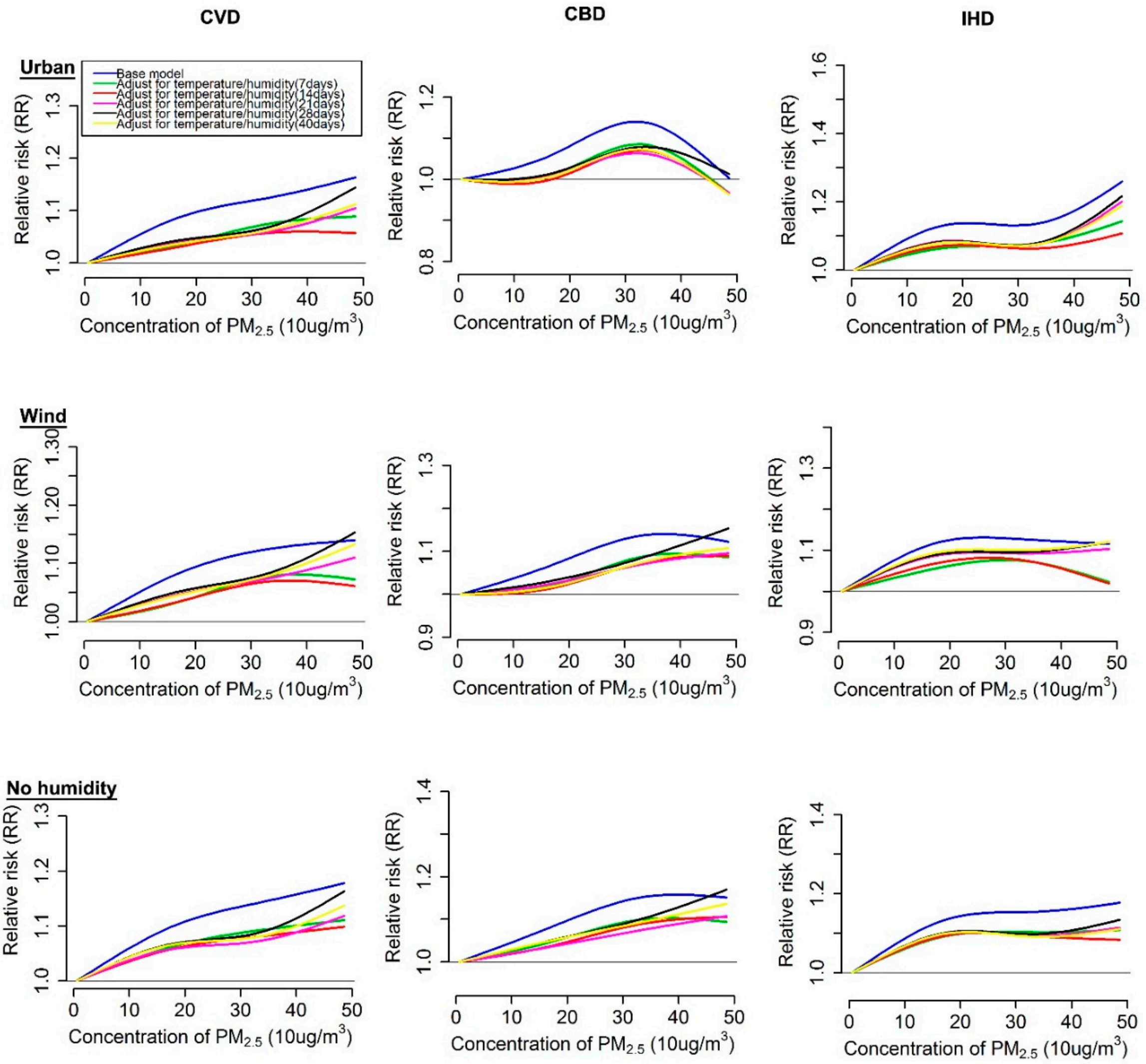

Figure S7. Exposure-response relationships for $PM_{2.5}$ (lag 0–1) with cardiovascular (CVD), cerebrovascular (CBD), and ischemic heart disease (IHD) mortality from extensively adjusted models for the whole population, according different modelling strategies. "Urban" represent the analysis was restricted to the districts within the radius of USA embassy monitoring station. "Wind" represent the effect of wind was additionally controlled for two days using natural cubic splines. "No-humidity" represent the humidity was not extensively controlled in models but with smoothed terms of two days mean using natural cubic splines. Values are estimated relative risk of mortality associated with $PM_{2.5}$ relatives to lowest value (5.83 μg/m^3). Base model (blue lines) adjusted effects of temperature and humidity only for two days, whereas the green, red, pink, black and yellow lines represent relationships from models with extensively adjusted cumulative effects of temperature and humidity for 7, 14, 21, 28 and 40 days, respectively, except for the "No humidity" row of picture, where the model only had extensive adjustments for temperature.

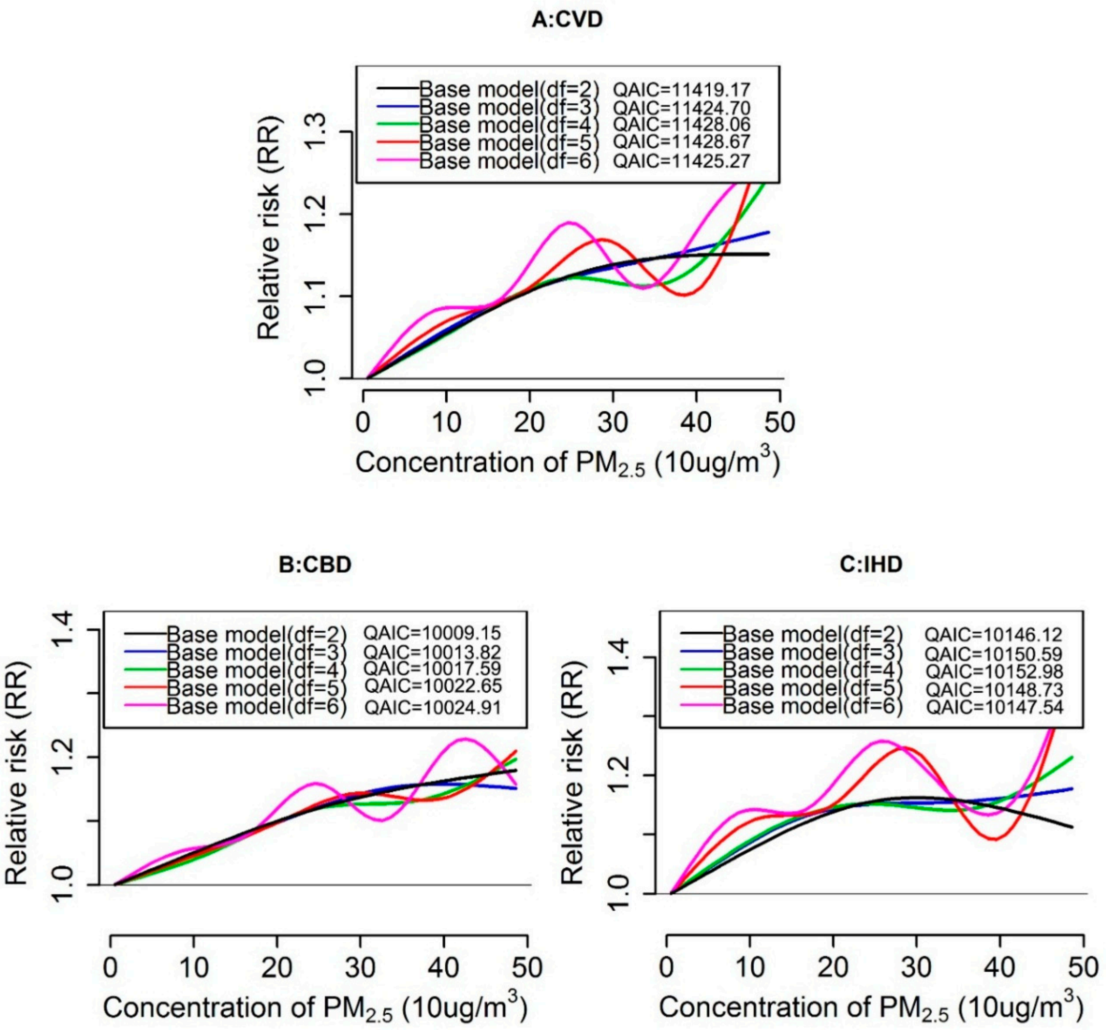

Figure S8. Cumulative exposure-response relationship between $PM_{2.5}$ and cardiovascular (CVD), cerebrovascular (CBD) and ischemic heart disease (IHD) mortality from the base model with different DF for a natural cubic spline in the $PM_{2.5}$ exposures space. The QAIC is the quasi-AIC for the quasi-Poisson regression. The reference level of $PM_{2.5}$ was 5.83 μg/m^3.

Table S2. Comparison of the mortality effect of $PM_{2.5}$ on cardiovascular events in studies in developed countries and China (estimates of $PM_{2.5}$ were showed as percent increase in risk and corresponded 95%CI per 10 μg/m^3 increase in concentration).

Authors	Location	Design	Lag	Temperature/Humidity Control		CVD	IHD (MI)	CBD (Stroke)
				Lag	Terms			
Kan et al., [3]	Shanghai, China	Time series	Lag 01	Lag 0	ps (temperature, 3) ps (humidity, 3)	0.41 (0.01, 0.82)	-	--
Ma et al., [4]	Shenyang, China	Time-stratified Case-crossover	Lag 01	Lag 0	ns (temperature, 3) ns (humidity, 3)	0.53 (0.09, 0.97)	-	-
Yang et al., [5]	Guangzhou, China	Time-stratified Case-crossover	Lag 01	Lag 0	ns (temperature, 3) ns (humidity, 3)	1.22 (0.63, 1.80)	-	-
Geng et al., [6]	Shanghai, China	Time series	Maximum lag of 3 days: based on polynomial distributed lag model	Maximum lag of 3 days	Tow dimension natural cubic spline with for temperature/humidity and lag days	0.78 (0.1, 1.43)	-	-
Huang et al., [7]	Xi'an, China	Time series	Lag 1–2	Lag 0	ns (temperature, 3) ns (humidity, 3)	0.27 (0.08, 0.46)	-	-
Xie et al., [8]	Beijing, China	Time series	Lag 0	Lag 0	ps (temperature, 6)	-	0.25 (0.10, 0.40)	-
			Lag 02	Lag 02	ps (temperature, 6)	-	0.34 (0.14, 0.53)	-
			Lag 04	Lag 04	ps (temperature, 6)	-	0.26 (0.03, 0.49)	-
Zanobetti and Schwartz [9]	112 cities, USA	Time series	Lag 01	Lag 01	ns (temperature, 3)	0.85 (0.46, 1.24)	1.18 (0.48, 1.89)-MI	1.78 (0.96, 2.62)-stroke
Franklin et al., [10]	27 communities, USA	Time-stratified case-crossover	Lag 1	Lag 01	Bs (apparent temperature, 3)	0.94 (−0.14, 2.02)	--	1.03 (0.02, 2.04)-stroke
Ostro et al., [11]	9 counties, California, USA	Time series	Lag 01	Lag 1	ns (temperature, 3) ns (humidity, 3)	0.60 (0.0, 1.1)	-	-
Samoli et al., [12]	12 cities, Europe	Time series	Lag 0–5	Lag 01	ns (temperature, 3)	0.86 (0.15, 1.57)	-	-

Note: Disease: CVD (cardiovascular disease), CBD (cerebrovascular disease), MI (myocardial infarction), IHD (ischemic heart disease). Single lag: lag 0 (current day), the rest lags were defined in the similar way; Multi-lags (moving average): lag 01 (two day means), the rest lags were defined in the similar way; Multi-lags (cumulative): lag 0–5 (current and previous five days), the rest lags were defined in the similar way; Splines: ns (natural cubic spline), ps (penalized spline), bs (quadratic spline).

Table S3. Spearman correlation coefficients between ambient pollutants and meteorological factors #.

	Temperature	Relative Humidity	Barometric Pressure	Wind
$PM_{2.5}$	0.17 *	0.58 *	−0.27 *	−0.47 *
Temperature		0.39 *	−0.87 *	0.00
Relative humidity			−0.38 *	−0.46 *
Barometric pressure				−0.03

# $PM_{2.5}$ was measured as 10 ug/m^3, temperature was measured as °C, relative humidity was measured as %, barometric pressure was measured as kPa and wind was measured as m/s.
* p-value < 0.05.

Table S4. Sensitivity analysis for the associations between three type of cardiovascular disease mortality and $PM_{2.5}$ according to different model choices *.

Outcomes	Adjusted Days [a]	Primary Results	Choices of the Selected Strata [b]			Adjusted for Wind [c]	Restricted Districts [d]	Only for Temperature [e]
			Strata-21days	Strata-30days	Stratum			
CVD	2	0.42 (0.28, 0.56)	0.31 (0.17, 0.46)	0.46 (0.32, 0.60)	0.44 (0.29, 0.59)	0.36 (0.21, 0.51)	0.37 (0.22, 0.53)	0.36 (0.21, 0.51)
	7	0.25 (0.11, 0.40)	0.21 (0.06, 0.36)	0.27 (0.13, 0.42)	0.28 (0.12, 0.44)	0.22 (0.07, 0.37)	0.21 (0.05, 0.38)	0.23 (0.08, 0.38)
	14	0.22 (0.07, 0.37)	0.18 (0.03, 0.33)	0.22 (0.06, 0.37)	0.26 (0.10, 0.42)	0.19 (0.04, 0.35)	0.16 (0.00, 0.33)	0.21 (0.06, 0.36)
	21	0.24 (0.09, 0.38)	0.18 (0.03, 0.33)	0.20 (0.05, 0.35)	0.28 (0.12, 0.44)	0.22 (0.07, 0.38)	0.18 (0.02, 0.35)	0.20 (0.05, 0.35)
	28	0.27 (0.13, 0.42)	0.24 (0.09, 0.39)	0.25 (0.11, 0.40)	0.31 (0.16, 0.47)	0.25 (0.10, 0.40)	0.22 (0.05, 0.38)	0.25 (0.10, 0.40)
	40	0.25 (0.11, 0.39)	0.21 (0.07, 0.35)	0.24 (0.10, 0.38)	0.29 (0.14, 0.44)	0.24 (0.10, 0.40)	0.19 (0.04, 0.35)	0.22 (0.08, 0.38)
CBD	2	0.42 (0.23, 0.62)	0.27 (0.07, 0.47)	0.41 (0.22, 0.60)	0.43 (0.23, 0.64)	0.38 (0.18, 0.58)	0.36 (0.14, 0.58)	0.38 (0.18, 0.58)
	7	0.26 (0.05, 0.47)	0.17 (−0.04, 0.39)	0.25 (0.04, 0.46)	0.27 (0.05, 0.50)	0.24 (0.03, 0.46)	0.21 (−0.03, 0.45)	0.26 (0.03, 0.46)
	14	0.22 (0.01, 0.43)	0.15 (−0.07, 0.36)	0.19 (−0.02, 0.40)	0.24 (0.02, 0.47)	0.21 (−0.01, 0.42)	0.17 (−0.07, 0.41)	0.23 (0.03, 0.44)
	21	0.21 (0.00, 0.42)	0.15 (−0.06, 0.37)	0.18 (−0.04, 0.39)	0.23 (0.00, 0.45)	0.20 (−0.01, 0.42)	0.15 (−0.08, 0.40)	0.21 (−0.01, 0.42)
	28	0.27 (0.06, 0.47)	0.23 (0.02, 0.44)	0.23 (0.02, 0.44)	0.28 (0.06, 0.49)	0.25 (0.04, 0.47)	0.20 (−0.04, 0.44)	0.28 (0.07, 0.49)
	40	0.23 (0.03, 0.42)	0.18 (−0.02, 0.39)	0.20 (0.00, 0.40)	0.25 (0.04, 0.45)	0.21 (0.00, 0.42)	0.17 (−0.06, 0.40)	0.25 (0.05, 0.46)
IHD	2	0.47 (0.26, 0.67)	0.38 (0.17, 0.59)	0.54 (0.34, 0.74)	0.50 (0.28, 0.73)	0.38 (0.16, 0.59)	0.44 (0.22, 0.66)	0.38 (0.16, 0.59)
	7	0.27 (0.05, 0.49)	0.23 (0.01, 0.45)	0.30 (0.08, 0.52)	0.30 (0.07, 0.54)	0.20 (−0.02, 0.43)	0.24 (0.00, 0.47)	0.23 (0.02, 0.45)
	14	0.25 (0.03, 0.47)	0.22 (0.00, 0.45)	0.27 (0.04, 0.49)	0.31 (0.07, 0.56)	0.21 (−0.02, 0.44)	0.20 (−0.04, 0.44)	0.23 (0.01, 0.45)
	21	0.30 (0.08, 0.53)	0.24 (0.01, 0.46)	0.26 (0.03, 0.49)	0.36 (0.12, 0.60)	0.28 (0.05, 0.51)	0.26 (0.01, 0.50)	0.25 (0.02, 0.47)
	28	0.33 (0.11, 0.54)	0.28 (0.06, 0.51)	0.31 (0.09, 0.53)	0.38 (0.14, 0.61)	0.29 (0.07, 0.52)	0.28 (0.04, 0.51)	0.26 (0.04, 0.48)
	40	0.33 (0.12, 0.54)	0.30 (0.08, 0.51)	0.34 (0.13, 0.55)	0.37 (0.14, 0.59)	0.31 (0.09, 0.53)	0.26 (0.04, 0.49)	0.24 (0.02, 0.47)

* Estimates were measured as percent increase (95% CI) in risk of mortality per 10 μg/m³ increase in $PM_{2.5}$ concentration for the whole population. [a] The 2 days represent the effects of temperature and humidity were adjusted with smoothed terms for two days using natural cubic splines in base models, whereas the 7, 14, 21, 28 and 40days represent the extensively adjusted exposure days (lags) for temperature and humidity by introducing cross-basis terms in extensively adjusted models; [b] "Strata-21days" and "strata-30days" represent fixed and disjoined strata of 21 and 30days in the same year were used to select the control days, while the stratum present the control days were selected as the same day of the week in the same month and year as the case period. [c] Adjusted for two days mean of wind using natural cubic splines; [d] Districts within the radius of US embassy; [e] Only extensively adjusting for temperature based on extensively adjusted models.

Table S5. Sensitivity analysis of associations between $PM_{2.5}$ and mortality after controlling for temperature /humidity for 21days with different DF for temperature/humidity (from 3 to 6) space and lag-space (from 3 to 6) selected in the cross-basis function.

Temperature DF	Lag DF	CVD		CBD		IHD	
		**Percent Increase (95% CI)**	**QAIC**	**Percent Increase (95% CI)**	**QAIC**	**Percent Increase (95% CI)**	**QAIC**
3	3	0.28 (0.15, 0.40)	11,119.62	0.26 (0.09, 0.44)	9820.693	0.35 (0.17, 0.53)	9945.625
3	4	0.24 (0.09, 0.38)	11,097.70	0.21 (0.00, 0.42)	9812.629	0.31 (0.09, 0.53)	9948.058
3	5	0.22 (0.07, 0.37)	11,104.29	0.20 (−0.02, 0.41)	9821.728	0.28 (0.05, 0.50)	9952.617
3	6	0.20 (0.05, 0.35)	11,101.59	0.19 (−0.02, 0.40)	9828.724	0.26 (0.03, 0.48)	9954.099
4	3	0.28 (0.16, 0.40)	11,051.26	0.27 (0.10, 0.44)	9792.616	0.34 (0.16, 0.52)	9906.615
**4**	**4**	**0.24 (0.09, 0.38)**	**11,026.27**	**0.21 (0.00, 0.42)**	**9784.822**	**0.30 (0.08, 0.53)**	**9912.469**
4	5	0.22 (0.07, 0.37)	11,036.21	0.20 (−0.01, 0.41)	9797.179	0.27 (0.05, 0.50)	9920.629
4	6	0.20 (0.05, 0.35)	11,036.29	0.19 (−0.02, 0.41)	9808.397	0.25 (0.02, 0.47)	9924.297
5	3	0.27 (0.15, 0.39)	11,048.40	0.26 (0.09, 0.43)	9793.110	0.33 (0.15, 0.51)	9910.304
5	4	0.22 (0.08, 0.37)	11,027.05	0.21 (0.00, 0.42)	9787.866	0.28 (0.06, 0.50)	9921.519
5	5	0.21 (0.06, 0.36)	11,039.79	0.19 (−0.02, 0.41)	9804.825	0.25 (0.02, 0.47)	9932.496
5	6	0.20 (0.04, 0.35)	11,037.83	0.20 (−0.02, 0.41)	9816.247	0.23 (0.00, 0.45)	9937.068
6	3	0.28 (0.16, 0.41)	11,052.95	0.27 (0.10, 0.44)	9803.815	0.35 (0.17, 0.53)	9914.233
6	4	0.24 (0.08, 0.39)	11,037.55	0.21 (0.00, 0.43)	9802.293	0.29 (0.07, 0.52)	9932.013
6	5	0.22 (0.07, 0.37)	11,055.52	0.20 (−0.01, 0.42)	9822.154	0.26 (0.03, 0.48)	9948.286
6	6	0.20 (0.05, 0.35)	11,054.75	0.20 (−0.02, 0.42)	9835.935	0.23 (0.00, 0.46)	9956.010

Note: Estimates were percent increase in risk of mortality of cardiovascular (CVD), cerebrovascular (CBD) and ischemic heart disease (IHD) for 10 $\mu g/m^3$ increase in $PM_{2.5}$. QAIC is the Quasi-AIC, which always used to assess the modeling fitting of Quasi-Poisson regression model. Results of present study with DF of 4 for both temperature/humidity space and lag-space were labeled as black bold.

References

1. Junger, W.L.; de Leon, A.P. Imputation of missing data in time series for air pollutants. *Atmos. Environ.* **2015**, *102*, 96–104.
2. Hastie, T.; Tibshirani, R. *Generalized Additive Model*; Chapman and Hall: London, UK, 1990; pp. 297–310.
3. Kan, H.D.; London, S.J.; Chen, G.H.; Zhang, Y.H.; Song, G.X.; Zhao, N.Q.; Jiang, L.L.; Chen, B.H. Differentiating the effects of fine and coarse particles on daily mortality in shanghai, china. *Environ. Int.* **2007**, *33*, 376–384.
4. Ma, Y.J.; Chen, R.J.; Pan, G.W.; Xu, X.H.; Song, W.M.; Chen, B.H.; Kan, H.D. Fine particulate air pollution and daily mortality in Shenyang, China. *Sci. Total Environ.* **2011**, *409*, 2473–2477.
5. Yang, C.X.; Peng, X.W.; Huang, W.; Chen, R.J.; Xu, Z.C.; Chen, B.H.; Kan, H.D. A time-stratified case-crossover study of fine particulate matter air pollution and mortality in Guangzhou, China. *Int. Arch. Occup. Environ. Health* **2012**, *85*, 579–585.
6. Geng, F.H.; Hua, J.; Mu, Z.; Peng, L.; Xu, X.H.; Chen, R.J.; Kan, H.D. Differentiating the associations of black carbon and fine particle with daily mortality in a Chinese city. *Environ. Res.* **2013**, *120*, 27–32.
7. Huang, W.; Cao, J.J.; Tao, Y.B.; Dai, L.Z.; Lu, S.E.; Hou, B.; Wang, Z.; Zhu, T. Seasonal variation of chemical species associated with short-term mortality effects of $PM_{2.5}$ in Xi'an, a central city in China. *Am. J. Epidemiol.* **2012**, *175*, 556–566.
8. Xie, W.X.; Li, G.; Zhao, D.; Xie, X.Q.; Wei, Z.H.; Wang, W.; Wang, M.; Li, G.X.; Liu, W.R.; Sun, J.Y.; et al. Relationship between fine particulate air pollution and ischaemic heart disease morbidity and mortality. *Heart* **2015**, *101*, 257–263.
9. Zanobetti, A.; Schwartz, J. The effect of fine and coarse particulate air pollution on mortality: A national analysis. *Environ. Health Perspect.* **2009**, *117*, 898–903.
10. Franklin, M.; Zeka, A.; Schwartz, J. Association between $PM_{2.5}$ and all-cause and specific-cause mortality in 27 US communities. *J. Expo. Sci. Env. Epidemiol.* **2007**, *17*, 279–287.
11. Ostro, B.; Broadwin, R.; Green, S.; Feng, W.Y.; Lipsett, M. Fine particulate air pollution and mortality in nine california counties: Results from calfine. *Environ. Health Perspect.* **2006**, *114*, 29–33.
12. Samoli, E.; Stafoggia, M.; Rodopoulou, S.; Ostro, B.; Declercq, C.; Alessandrini, E.; Diaz, J.; Karanasiou, A.; Kelessis, A.G.; Le Tertre, A.; et al. Associations between fine and coarse particles and mortality in mediterranean cities: Results from the med-particles project. *Environ. Health Perspect.* **2013**, *121*, 932–938.

© 2016 by the authors; licensee MDPI, Basel, Switzerland. This article is an open access article distributed under the terms and conditions of the Creative Commons by Attribution (CC-BY) license (http://creativecommons.org/licenses/by/4.0/).
